# Angiomatous pleomorphic xanthoastrocytoma: a case report and literature review

**DOI:** 10.1186/s13000-016-0524-0

**Published:** 2016-08-09

**Authors:** Yue-Feng Jiang, Yang Liu, Ye-Lin Wang, Hong-Yi Cao, Liang Wang, Hong-Tao Xu, Qing-Chang Li, Xue-Shan Qiu, En-Hua Wang

**Affiliations:** 1 Department of Pathology, the First Affiliated Hospital and College of Basic Medical Sciences, China Medical University, Shenyang, 110001 China; 2 Institute of Pathology and Pathophysiology, China Medical University, Shenyang, 110001 China

**Keywords:** Pleomorphic xanthoastrocytoma, Angiomatous variant, BRAF mutation

## Abstract

**Background:**

Pleomorphic xanthoastrocytoma is rare, accounting for <1 % of all central nervous system (CNS) neoplasms. Angiomatous pleomorphic xanthoastrocytoma is an extremely rare variant of pleomorphic xanthoastrocytoma, with only six cases reported thus far.

**Case presentation:**

A 24-year-old Chinese female patient who presented with seizure and loss of consciousness for 15 min underwent computed tomography and magnetic resonance imaging, which revealed a mass in the left parietal lobe. Histologically, the tumor was characterized by pleomorphic tumor cells and prominent vascularity. The angiomatous region varied, ranging from a sinusoidal pattern to a venous malformation. Focal fibrinoid necrosis, hyalinization, and a moderate infiltration by lymphocytes and plasma cells were visible in the vessel wall. The tumor cells were in close proximity with adjacent small vessels. Capillaries adjacent to or extending between tumor cells were focally observed. Most tumor cells were positive for glial fibrillary acidic protein and oligodendrocyte lineage transcription factor 2. The Ki-67 index was low. Based on the patient’s history, clinical data, and pathological findings, she was diagnosed with angiomatous pleomorphic xanthoastrocytoma (WHO grade II).

**Conclusions:**

This case serves as a reminder to pathologists of the need to be aware of this rare variant of pleomorphic xanthoastrocytoma to avoid a misdiagnosis of this indolent CNS tumor and therefore inappropriate treatment.

## Background

Pleomorphic xanthoastrocytoma (PXA) is an uncommon tumor of the central nervous system (CNS). It was first described as a unique entity in 1979 [1]. PXA typically develops in children and young adults, with no predilection for males vs. females. It usually develops in the superficial cortex, especially in the temporal lobes, and meningeal involvement is common [2, 3]. Uncommon sites of PXA include the cerebellum [4, 5], ventricle [6, 7], spinal cord [8, 9], sella [10], retina [9, 11] and pineal gland [11–14]. Patients usually present with a prolonged history of seizure. Computed tomography (CT) and magnetic resonance imaging (MRI) reveal either a cystic mass with an enhancing mural nodule or a solid mass. Histologically, PXA is characterized by bizarre cytologic features and “lipidized” tumor cells with a foamy, lipid-laden cytoplasm. These cells are seen in approximately 25 % of cases. However, most PXAs are composed of spindle-shaped cells with astrocytic features in storiform or fascicular array admixed with tumor giant cells that display worrisome, often severe, nuclear abnormalities. Intranuclear inclusions, eosinophilic granular bodies (EGB), and perivascular lymphocytes are often present. In general, these tumors have a very low mitotic rate and microvascular proliferation or necrosis is rare. Thus, histologically, most PXAs are WHO grade II. On occasion, however, a PXA will have a high mitotic rate (>5 mitoses per 10 high-power fields) and areas of necrosis, which together are features of anaplastic astrocytoma. These tumors, designated anaplastic PXA, WHO grade III instead of “PXA with anaplastic features”, have been added to the 2016 CNS WHO as a distinct entity. Compared with PXA (WHO grade II), anaplastic PXA (WHO grade III) is associated with an aggressive behavior and the survival of affected patients is poor [15]. Variant forms in which PXA exhibits a mixed histologic pattern have been described and include “composite” tumors, harboring gangliogliomatous and xanthoastrocytomatous components, and PXAs with uncommon histological features, such as a cohesive, nesting, or alveolar growth pattern [16], a hyalinizing, angiomatous pattern [17], or a melanotic pigmentation [18, 19]. The unusual histological appearance of PXA can complicate its diagnosis. Moreover, these tumors may be misdiagnosed if the pathologist is not familiar with the full spectrum of their variations. To improve clinical and pathological knowledge of these tumors, we present a new case of angiomatous pleomorphic xanthoastrocytoma and provide a review of previously published cases.

## Case presentation

### Clinical history

**Fig. 1:**
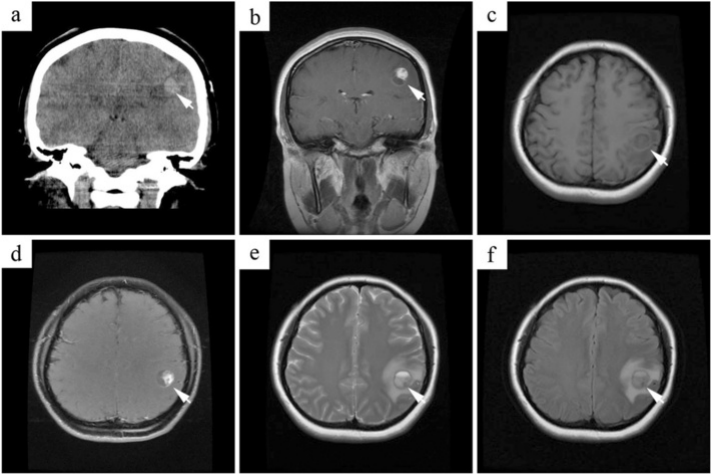
Imaging examination results. **a** Contrast-enhanced CT reveals a hyperintense oval mass in the left parietal lobe. Post-contrast T1-weighted MRI (**b** coronal view), T1-weighted MRI (**c** horizontal view), post-contrast T1-weighted MRI (**d** horizontal view), T2-weighted MRI (**e** horizontal view), and FLAIR (**f** horizontal view) show a well-circumscribed, partially cystic mass with a focally enhancing mural nodule and minimal surrounding edema (both indicated by arrows) as well as a mass effect in the left parietal lobe

A 24-year-old female was admitted to our hospital. One week earlier, she had suffered a seizure that had caused a loss of consciousness lasting 15 min. She did not have headache, vomiting, visual disturbance, or hypoacusis. Her family history was not remarkable. Neurological examination revealed no abnormalities. Contrast-enhanced CT (Fig. 1a) and post-contrast T1-weighted MRI (Fig. 1b) showed a hyperintense oval mass in the left parietal lobe. Its largest dimension was 1.4 cm. T1-weighted (Fig. 1c), post-contrast T1-weighted (Fig. 1d), T2-weighted (Fig. 1f), and fluid-attenuated inversion recovery (FLAIR) (Fig. 1e) MRI showed a well-circumscribed, partially cystic mass with a focally enhancing mural nodule, minimal surrounding edema, and a mass effect in the left parietal lobe. Neuroendoscopic excision performed through a parietal hole revealed a lesion with cystic and solid components and a red-meat color in the left parietal lobe. The mass was well demarcated and adhered slightly to the surrounding normal tissue. A tumor 1.9 × 2 × 2.1 cm in size was completely resected. After 10 months of follow-up, the patient was alive with no tumor recurrence or metastasis and good seizure control.

### Materials and methods

The tumor tissues were fixed in 10 % formalin and embedded in paraffin. Sections (4 μm) were cut from each paraffin block; one was stained with H&E and the others were used in immunohistochemistry (IHC) analyses. IHC staining was performed using the streptavidin-peroxidase system (Ultrasensitive; Mai Xin Inc., Fuzhou, China), according to the manufacturer’s instructions, and commercially available prediluted monoclonal antibodies against the following antigens: epithelial membrane antigen (EMA), pan-cytokeratin (AE1/AE3), vimentin, glial fibrillary acidic protein (GFAP), oligodendrocyte lineage transcription factor 2 (olig2), NeuN, synaptophysin, isocitrate dehydrogenase 1 (IDH1), CD31, CD34, S100, neurofilament protein (NF), p53, CD68, inhibin-α, D2-40, and Ki-67. For the negative controls, the primary antibody was replaced with PBS.

#### BRAF^V600E^ Mutation Analysis

The CFDA-approved human BRAF^V600E^ ARMSPCR kit (Amoy Diagnostics Co. Ltd., Xiamen, China) was used to detect the BRAF^V600E^ mutation. The quality of the extracted DNA was confirmed based on amplifications of a housekeeping gene and its analysis in the kit’s HEX channel, as recommended by the manufacturer. The amplification protocol consisted of 47 cycles (one cycle of 95 °C for 5 min; 15 cycles of 95 °C for 25 s, 64 °C for 20 s, and 72 °C for 20 s; and 31 cycles of 93 °C for 25 s, 60 °C for 35 s, and 72 °C for 20 s). FAM and HEX signals were collected during the third stage. The run files were analyzed and interpreted as specified by the manufacturer.

### Microscopic features

**Fig. 2:**
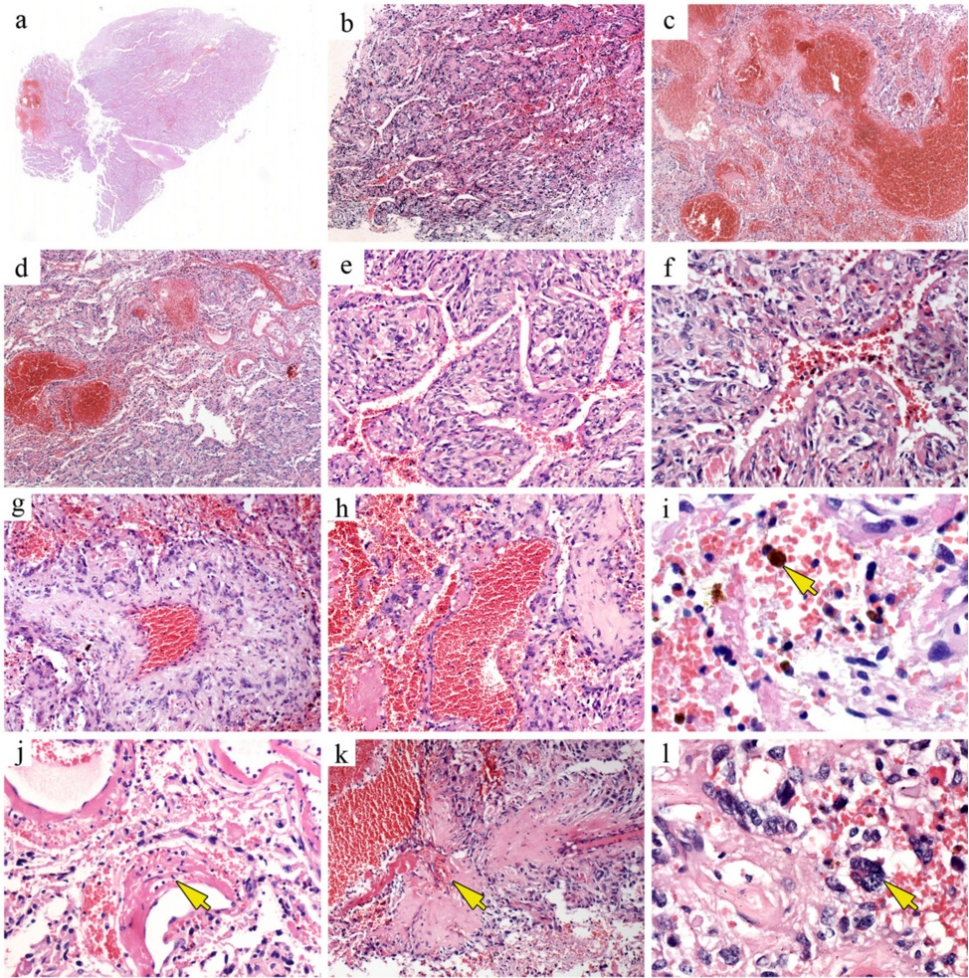
Histological features of angiomatous pleomorphic xanthoastrocytoma. **a**–**d** The entire neoplasm is invested with an abundant vascular meshwork consisting of a sinusoidal configuration and venous malformation. **e**, **f** The tumor is composed of neoplastic astrocytes surrounded by a poorly canalized configuration. **g**, **h** Foci of abnormal veins of varying size are observed within the tumor. The blood vessel walls are of variable thickness and some show hyaline degeneration; others are large, thin-walled vessels with irregular lumens. **i** Foci of hemosiderin are present within the tumor. **j** Focal fibrinoid necrosis, hyalinization, and a moderate infiltration of lymphocytes and plasma cells are observed in the vessel wall. **k** A desmoplastic reaction caused by plasma proteins that have exuded through the leaky walls of newly formed blood vessels. **l** The pleomorphic histology of the tumors includes a varying cell density mainly consisting of cells with significant nuclear and cellular pleomorphism. The corresponding histological features are indicated by arrows

**Fig. 3:**
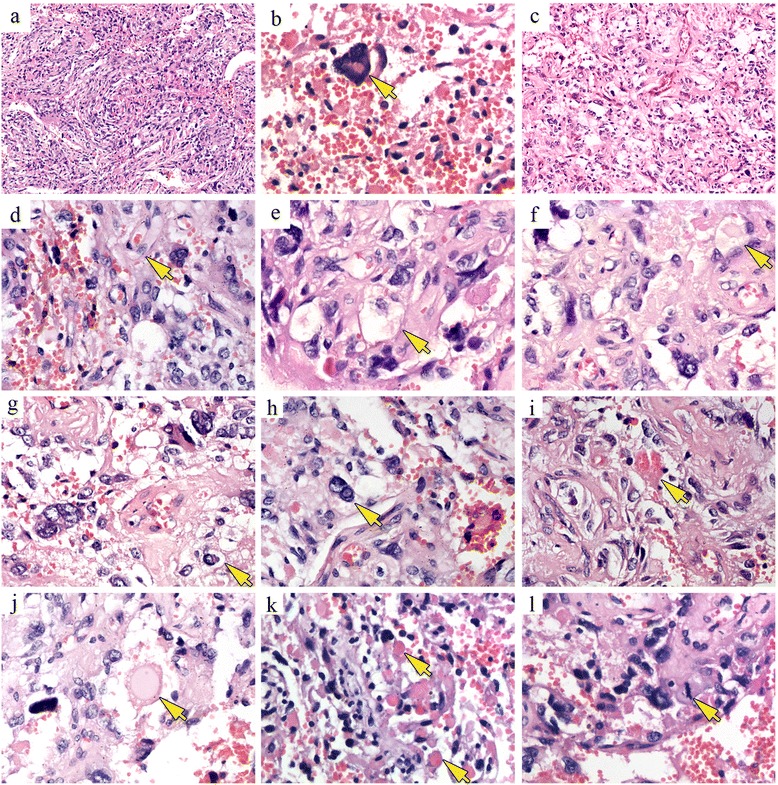
Histological features of angiomatous pleomorphic xanthoastrocytoma. **a** Spindle-shaped cells, arranged in fascicular and fibrillary patterns, are observed in focal areas. **b**, **c** Calcification and microcystic formation are present among the tumor cells. **d** In some areas, the small vessels and neoplastic astrocytes are in close proximity to each other, with capillaries adjacent to or extending between the tumor cells. **e**–**g** The cells include mono- or multinucleated giant astrocytes with a foamy or vacuolated cytoplasm. **h** Focal clusters of small lymphocytes and intranuclear inclusions are also evident. **i**–**k** EGB (intensely eosinophilic or pale) and eosinophilic hyaline droplets between tumor cells. **l** Less than one mitosis per high-power field. In each figure part, the corresponding histological features are indicated by arrows

**Fig. 4:**
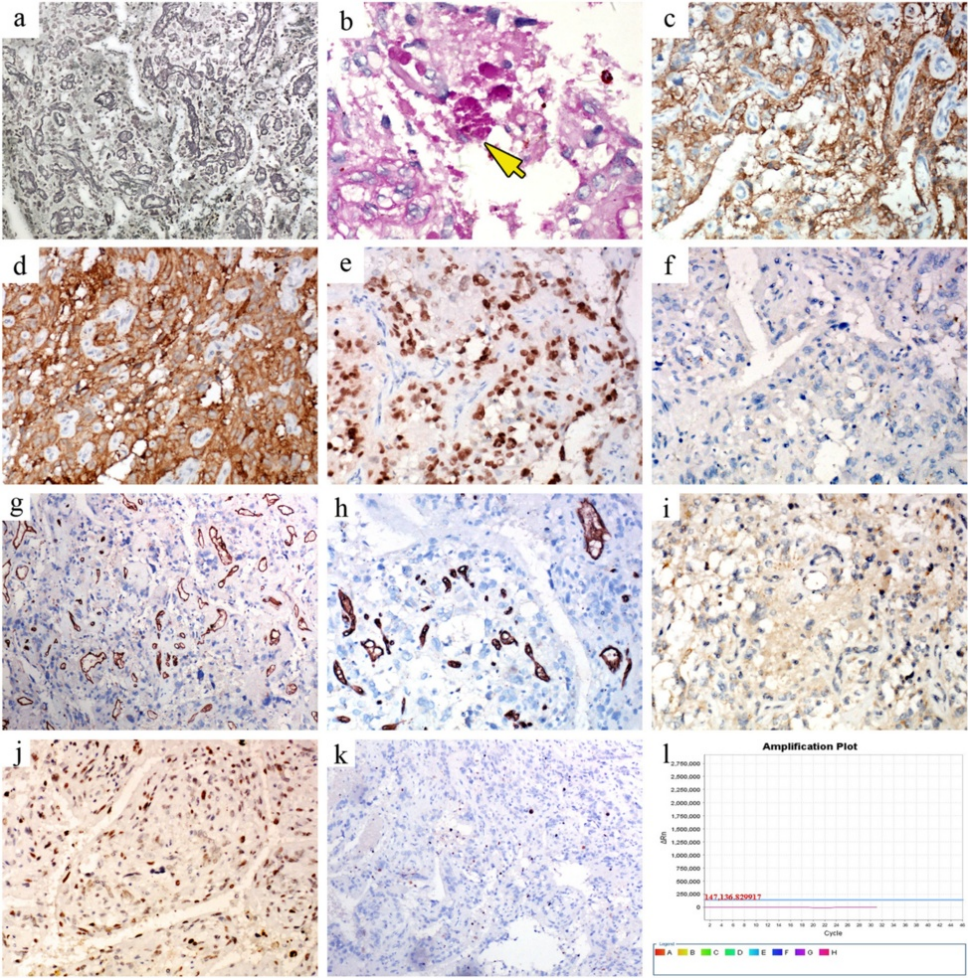
Immunohistochemical and specific staining, and BRAFV600E mutation analysis. **a** Silver staining shows reticulin fibers encircling the blood vessels, but they are hardly present among tumor cells. **b** The EGBs stain red with PAS (Fig. 4b). **c**–**f** The tumor cells are diffusely positive for GFAP, S100, and olig2, but negative for EMA. **g**, **h** The tumor cells are negative but the capillaries within the tumor are positive for CD34. **i** The tumor cells are negative for IDH1. **j** Approximately 50 % of the tumor cell nuclei stain positively for p53. **k** The Ki-67 labeling index is approximately 2 %. **l** The tumor was negative for a BRAF^V600E^ mutation. In each figure part, the corresponding histological features are indicated by arrows

Histologically, the tumor was characterized by markedly pleomorphic tumor cells and a highly vascular configuration. At low magnification, the entire neoplasm was invested with an abundant vascular meshwork characterized by a sinusoidal configuration and venous malformation (Fig. 2a–d). The neoplastic astrocytes that composed the tumor were surrounded by a poorly canalized configuration (Fig. 2e, f). Foci of abnormal veins of varying sizes were present within the tumor (Fig. 2g, h). The walls of these blood vessels were of variable thickness; some were thickened and showed hyaline degeneration (Fig. 2g); others were large, thin-walled vessels with irregular lumens (Fig. 2h). There was evidence of both acute and chronic hemorrhage, with foci of hemosiderin (Fig. 2i). Other features of the vessel walls were focal fibrinoid necrosis, hyalinization, and a moderate infiltration of lymphocytes and plasma cells (Fig. 2j). The presence of fibrosis or a desmoplastic reaction suggested the secondary organization of plasma proteins that had exuded through the leaky walls of the newly formed blood vessels (Fig. 2k). Together, these features suggested a hemangioma. However, the sections showed the pleomorphic histology of the tumor, with a varying cell density that, at high magnification, consisted mainly of cells with significant nuclear and cellular pleomorphism (Fig. 2l). The spindle-shaped cells, arranged in fascicular and fibrillary patterns, occurred focally (Fig. 3a). Foci of calcification (Fig. 3b) and microcystic formation (Fig. 3c) were also seen among the tumor cells, which were in close proximity with the adjacent small blood vessels. Capillaries adjacent to or protruding into the tumor cell cytoplasm were detected focally (Fig. 3d), together with mono- or multinucleated astrocytes with a foamy or vacuolated cytoplasm (Fig. 3e–g); however, typical giant xanthoastrocytes were not observed in this case. Focal clusters of small lymphocytes with intranuclear inclusions (Fig. 3h) were also evident. EGB (intensely eosinophilic or pale) and eosinophilic hyaline droplets were also observed among the tumor cells (Fig. 3i–k). Despite the focal marked pleomorphism, pseudo-palisading necrosis was not present. Mitoses were < 1 per 10 high-power field (Fig. 3l), but atypical mitoses were absent. Silver staining revealed reticulin fibers encircling the blood vessels, but they were rare among the tumor cells (Fig. 4a). The EGBs stained red with periodic acid-Schiff (PAS) stain (Fig. 4b).

### Immunohistochemistry

Immunohistochemically, the tumor cells were diffusely positive for GFAP (Fig. 4c), S100 (Fig. 4d), olig2 (Fig. 4e), synaptophysin, and vimentin, but negative for EMA (Fig. 4f), CD31, CD34 (Fig. 4g, h), NeuN, inhibin-α, D2-40, and IDH1 (Fig. 4i). Some areas of the tumor stained positively for NF. Although the foamy macrophages around the tumor cells stained positively for CD68, the tumor cells were completely negative. Positive nuclear staining for p53 was detected in ~50 % of the tumor cells (Fig. 4j). The Ki-67 labeling index was approximately 2 % (Fig. 4k).

### BRAF^V600E^ mutation analysis

Two recent studies, including in pediatric and in adult patients, suggested a relationship of PXA to the BRAF^V600E^ mutation. However, the mutation was not detected in our patient (Fig. 4l).

## Discussion

Angiomatous PXA was first described by Sugita et al. in 1990 [17]. To date, the five reports in the English-language literatures describe six cases [17, 20–23]. Our patient represents the seventh case. Given the rarity of PXA, we discuss this case in the context of a literature review of the previously reported cases. The clinical and follow-up data of these cases and of the current one are summarized in Table 1. Most patients with PXA have a history of epileptic seizures. None of the patients experienced tumor recurrence or metastasis during the follow-up period.

**Table 1:** Clinical data of reported angiomatous PXA

Case	Authors	A/G	Tumor location	Image finds	Symptoms	Follow-up
1	Sugita et al. 1990 [17]	19/F	ND	ND	1-year history of epileptic seizures	10 years, NOR
2	Sugita et al. 1990 [17]	26/M	ND	ND	6-month history of epileptic seizures	ND
3	Takahabashi et al. 1995 [23]	58/F	ND	ND	30-year history of epileptic seizures	2 years, NOR
4	Lee et al. 1996 [22]	45/M	left temporo-occipital lobe	cystic mass	a 15-year history of generalized tonic-clonic seizures	ND
5	Sugita et al. 1999 [20]	43/M	right temporal lobe	cystic-solid mass	a 20-year history of generalized epileptic seizures	one year, NOR
6	Richard et al. 1999 [21]	27/M	right medial frontal lobe	cystic-solid mass	3-year history of intermittent, transient, mild, left-sided weakness	1 year, NOR
7	Present case	24/F	left parietal lobe	cystic-solid mass	1-week history of epileptic seizures	10 months, NOR

A/G, Age/gender; F, female; M, male; ND, no data; NOR, no evidence of recurrence

The histological features of angiomatous PXA are the presence of an abundant vasculature. In our patient, the blood vessel walls were of variable thickness and some showed hyaline thickening. The abundance of blood vessels in some areas was such that the tumor resembled a hemangioma. Other features were neoplastic astrocytes and tiny blood vessels in close proximity to each other, and histological features consistent with the original description of angiomatous PXA. However, some areas of the tumor consisted of epithelioid tumor cells arranged trabecularly and surrounded by sinusoidal configurations (Fig. 2b, e, f). These sinusoidal channels were not lined by endothelial cells and were filled with erythrocytes (Fig. 2e, f). Previous reports of angiomatous PXA did not include a description of this histological pattern, which is, however, seen in other variants of PXA such as epithelioid PXA [16]. In our patient, in some areas of the tumor these two histological patterns blended imperceptibly, demonstrating the potential overlap of these two histological types in the same tumor.

The histological features of the seven known cases of angiomatous PXA are summarized in Table 2. As suggested by the name of this variant, its highly vascular configuration mimics the pattern seen in highly vascularized or “angiomatous” meningiomas and is a common histological feature. Although the typical giant xanthomatous tumor cells were not observed in the tumor specimen from our patient, the detection of prominent multinucleated tumor cells, foci of tumor cells with a foamy or vacuolated cytoplasm, giant cells, and EGB together supported a diagnosis of PXA. Degenerative changes, such as granular bodies, microcystic changes, hyaline droplets, hyalinized blood vessel walls, hemosiderin deposition, and calcification, seen in the other six cases of PXA, were also present in the tumor of our patient. The degeneration characteristics of these tumors might be related to the indolent course of angiomatous PXA, as there has been no recurrence in any of the previously described patients.

**Table 2:** The histological features of angiomatous PXA

Histological features	Sugita et al. 1990 [17]		Takahabashi et al. 1995 [23]	Lee et al. 1996 [22]	Sugita et al. 1999 [20]	Richard et al. 1999 [21]	Present case
	Case 1	Case 2	Case 3	Case 4	Case 5	Case 6	Case 7
Highly vascular configuration	+	+	+	+	+	+	+
Microcystic formation	ND	ND	ND	ND	+	ND	+, focal
storiform or fascicular growth pattern with spindled cells	ND	ND		+	ND	+	+, focal
Anaplasic features	ND	ND	ND	-	-	-	-
“Epithelioid” cells	ND	ND	ND	-	-	ND	+
Pleomorphism	+	+	+	ND	+	+	+
Foamy or vacuolated cytoplasm	+	+	+	+	+	+	+
Giant cells	+	+	+	ND	+	+	+
Xanthomatous tumor cells	+	+	ND	ND	+	+	-
Dysplastic neurons	+	+	+	ND	+	+	-
Intranuclear inclusions	ND	ND	ND	ND	ND	ND	+
Calcification	ND	ND	ND	ND	+	+	+
Rosenthal fibers	ND	ND	ND	ND	ND	+	-
Eosinophilic hyaline droplets	ND	ND	ND	ND	ND	+	+
Granular bodies							
eosinophilic	+	+	+	+	+	+	+
Pale	ND	ND	ND	ND	ND	ND	+
Hemosiderin deposition	ND	ND	ND	+	-	+	+, focal
Perivascular lymphocytes infiltration	+	+	ND	+	+	+	+
Reticulin network	ND	ND	ND	+	-	-	-
Mitoses	rare	rare	rare	rare	rare	rare	rare
Necrosis	-	-	-	-	-	-	-
Endothelial proliferation	-	-	-	-	-	-	-

+: the corresponding feature exists; −: the corresponding feature does not exist; ND: no data

The histological features of these tumors suggest their correlation with vascular malformation. However, whether these vascular changes represent a chronic clinical course or tumor degeneration, thus predicting a relatively favorable biological behavior of the PXA, is unclear. In the case presented by Sugita et al., obvious calcification of the vascular walls and extensive fibrosis were features of the tumor [17]. Lee et al. reported numerous hemosiderin-laden macrophages in their PXA specimen [22]. The histological features of the tumors in our case were similar to those reported by Sugita and Lee, but there were fewer hemosiderin-laden macrophages and calcification did not involve the vascular walls, nor was it as extensive. These discrepancies suggest the slower growth of the tumors in the cases reported by those authors, as both patients had a long history of epileptic seizures.

Immunohistologically, the tumor cells in all seven angiomatous PXAs reported thus far consistently expressed GFAP, S100, and olig2. However, CD34 and NF expression is not a constant feature and only roughly half of the tumor cell nuclei stained positively for p53. Although the overexpression of p53 in glioma indicates a poor prognosis, the relevance of p53 in PXA remains to be clarified. In a molecular analysis of PXA by Tabouret et al. [24], a BRAF^V600E^ mutation was a common molecular characteristic. An association between BRAF-mutated PXA, reticulin fiber deposition, and CD34 expression was also described [25], while in our case, neither reticulin fiber deposition (Fig. 4a) nor CD34 expression (Fig. 4g, h) was observed, which precisely demonstrates this phenomenon from reverse. In the case reported by Yamada et al. [26], the tumor harbored both a BRAF^V600E^ mutation and an IDH1 R132H mutation, although the latter has been seldom identified in PXA. In our patient, BRAF^V600E^ mutation analysis and IDH1 R132H immunohistological staining yielded negative results (Fig. 4l, i).

Because of the prominent nuclear pleomorphism (Fig. 3e, f, g) and mesenchymal-like foci (Fig. 3a) in the tumor removed from our patient, the differential diagnosis will include giant-cell glioblastoma, gliosarcoma, ganglioglioma, and pilocytic astrocytoma. Unlike PXA, giant-cell glioblastoma and gliosarcoma typically have significant mitotic activity, microvascular proliferation, and pseudo-palisading necrosis. Neither significant mitotic activity nor pseudo-palisading necrosis were seen in the present case, and the Ki-67 labeling index was ~2 %, which ruled out a diagnosis of giant-cell glioblastoma or gliosarcoma. Despite the abundant vasculature, glomeruloid microvascular proliferation, characteristic of glioblastoma, was not seen in this case. In addition, EGBs were prominent and are typical of PXA, but not gliosarcoma. The cystic mass with a focally enhancing mural nodule, revealed by MRI, also supports a diagnosis of PXA rather than gliosarcoma. PXA and ganglioglioma may have overlapping clinical, radiologic, and histologic features and in rare cases the two co-exist, forming a composite neoplasm. However, ganglioglioma is less pleomorphic, has a more obvious neuronal component, and lacks lipidized astrocytes. In our patient, the presence in the tumor of neoplastic astrocytes with a foamy or vacuolated cytoplasm and the negative immunostaining result for NeuN did not support a diagnosis of ganglioglioma. The tumor cells in pilocytic astrocytoma may have bizarre, atypical, or pleomorphic nuclei but their typical biphasic pattern, consisting of bipolar and loose-textured multipolar cells, was not identified in this case. In addition, angiomatous PXA should be distinguished with hemangioblastoma based on the abundant vasculature and the tumor cells with foamy or vacuolated cytoplasm. However, in hemangioblastoma, the stromal cells often label for S-100, NSE, CD56, inhibin-α, and D2-40. Reactivity for GFAP, if present, is usually limited to entrapped astrocytes, thus seldom presents with diffuse form. In the current case, the immunophenotype (diffusely positive for olig2 and GFAP, inhibin-α-, D2-40-) ruled out a diagnosis of hemangioblastoma.

In general, PXA has a relatively indolent clinical course. However, Kepes et al. [27] reported several instances in which local recurrences that developed at varying intervals after surgery transitioned to a more malignant type of astrocytoma (anaplastic astrocytoma or glioblastoma). Weldon-Linne et al. [28] also reported that PXA may have a less favorable course, with aggressive, malignant transformation even after a prolonged period of indolence. While malignant evolution is the exception, its occasional occurrence warns against considering PXA as benign. Tabouret et al. [24] found that the BRAF^V600E^ mutation was a common molecular characteristic of PXA and suggested its predictive value for progression-free survival in adult patients. However, p53 overexpression rather than a BRAF^V600E^ mutation was detected in the tumor from our patient. Whether their absence indicated a relatively unfavorable biological behavior remains to be determined in the long-term follow-up of our patient.

## Conclusion

Our case report of angiomatous pleomorphic xanthoastrocytoma is meant to serve as a reminder to pathologists to be aware of this rare variant of PXA. The description provided herein and the review of the features of these tumors, based on previously published cases, should help to avoid a misdiagnosis of this typically indolent CNS tumor.

## Abbreviations

CNS, central nervous system; CT, computed tomography; EGB, eosinophilic granular bodies; EMA, epithelial membrane antigen; GFAP, glial fibrillary acidic protein; IDH1, Isocitrate dehydrogenase 1; IHC, immunohistochemistry; MRI, magnetic resonance imaging; NF, neurofilament; olig2, oligodendrocyte lineage transcription factor 2; PXA, Pleomorphic xanthoastrocytoma
